# A positive effect of cumulative intergroup threat on reproductive success

**DOI:** 10.1098/rspb.2023.1853

**Published:** 2023-11-15

**Authors:** Amy Morris-Drake, Benjamin Cobb, Julie M. Kern, Andrew N. Radford

**Affiliations:** ^1^ School of Biological Sciences, University of Bristol, 24 Tyndall Avenue, Bristol BS8 1TQ, UK; ^2^ School of Environmental and Rural Science, University of New England, Armidale 2351, New South Wales, Australia

**Keywords:** intergroup, outgroup, conflict, fitness, reproductive success, dwarf mongooses

## Abstract

Outgroup conflict is a powerful selective force across all social taxa. While it is well documented that individual outgroup contests can have a range of direct and indirect fitness consequences, the cumulative pressure of outgroup threats could also potentially impact reproductive success. Here, we use long-term life-history data from a wild population of dwarf mongooses (*Helogale parvula*) to investigate how intergroup interaction (IGI) rate might influence breeding and offspring survival. IGI rate did not predict the number of litters produced in a season or the inter-litter interval. Unexpectedly, IGI rate was positively associated with the number of pups alive three months after emergence from the breeding burrow. This was not due to a difference in how many pups emerged but because those in groups experiencing more IGIs had a higher survival likelihood post-emergence. Detailed natural observations revealed that both IGI occurrence and the threat of intergroup conflict led to more sentinel behaviour by adults, probably reducing the predation risk to young. Our results contrast the previously documented negative effects of outgroup interactions on reproductive success and highlight the need to assess cumulative threat, rather than just the impact of physical contests, when considering outgroup conflict as a social driver of fitness.

## 1. Introduction

From ants to primates, animal groups face a variety of threats from conspecific outsiders seeking valuable resources such as matings, breeding positions, food, sleeping sites and territories [1–6]. Threats may come from single individuals, coalitions or rival groups; we use ‘outgroup’ conflict to refer to conflict with any conspecific outsider(s) [5]. Outgroup conflict is recognized as a powerful evolutionary force [7–9], so fitness assessments are crucial to elucidate the selection pressures exerted. To date, most research in this regard has focused on the survival and reproductive consequences of single, physical contests (reviewed in [10,11]). For example, an aggressive interaction can result in loss of life (of participants or young), paternity (through extra-group matings) or breeding position (if the incumbent is usurped by an outsider) [2,12–14]. Moreover, there may be delayed fitness consequences of a contest if, for instance, participants injured in fights subsequently have reduced survival or breeding performance [15,16], or if a decrease in group size leads to a greater mortality risk from predation and starvation [17,18]. However, the influence of conspecific outsiders is unlikely to be restricted to individual confrontations between rivals; as with predators, there may also be cumulative effects from the overall threat [19].

The cumulative effects of living in a landscape of outgroup conflict are predicted to impact breeding and reproductive output [10]. For instance, if threats from outsiders generate chronic stress [20], this may lead to increased susceptibility to disease, reduced body condition and lessened investment in reproduction by adults [21–24]. Chronic stress in adults could also have knock-on consequences for offspring, through maternal effects or decreases in the quality of parental care [3,25,26]. However, research quantifying such cumulative fitness consequences of outgroup threat is rare. Using long-term observational data, two studies have found negative effects of an increased level of outgroup threat: in banded mongooses (*Mungos mungo*), pup survival was lower [27]; while in chimpanzees (*Pan troglodytes*), fetal survival was lower and inter-birth intervals were longer [28]. Similarly, recent experimental work with the daffodil cichlid (*Neolamprologus pulcher*) showed that elevated levels of outgroup threat resulted in longer inter-clutch intervals and the production of fewer and smaller offspring at one month post-hatching [29].

Here, we use long-term life-history and behavioural data from a wild population of dwarf mongooses (*Helogale parvula*) to investigate how outgroup conflict—specifically, the rate of intergroup interactions (IGIs)—might influence breeding and offspring survival, and to explore possible reasons for the relationships found. Intergroup conflict is the subset of outgroup conflict involving threats from rival groups or coalitions, rather than individual outsiders. Dwarf mongoose groups each defend a territory from conspecific outsiders, often engaging in contests with rival groups or coalitions when they are encountered [30,31]. As cooperative breeders, all group members contribute to a range of communal activities including pup care, territory defence and anti-predator vigilance [31,32]. By continuously monitoring a habituated study population in South Africa across multiple years [32,33], we accumulated long-term data that allowed testing of the predicted negative association between IGI rate and reproductive success. Having found an unexpected positive relationship between IGI rate and pup survival (see Results), we used detailed observations of sentinel behaviour—dwarf mongoose adults commonly adopt a raised position to scan for danger while groupmates continue with other activities such as foraging [32,34]—to consider whether elevated outgroup threat might drive increased vigilance, and thus be a potential explanation for greater offspring survival.

## 2. Methods

### (a) Study species

Dwarf mongooses are a cooperatively breeding species: the dominant pair monopolize reproduction, while subordinate group members of both sexes help raise the offspring of the dominant pair [35,36]. Dominant females generally produce up to three litters per breeding season. Subordinate females sometimes breed at the same time as dominants; of 101 litters produced by dominant females across six breeding seasons and 11 groups, a subordinate female gave birth around the same time as the dominant on a mean of 30% of occasions per season (range 7–47%). Each litter contains up to six pups [37]. Following birth, pups remain underground for around the first three weeks of life; they then stay in the vicinity of the burrow (within a few metres of it) for around three weeks post-emergence. Throughout this, around six week period, they are cared for by babysitters, who feed and protect the young from a range of aerial and terrestrial predators [38]. The babysitter changes throughout the day to ensure all adult individuals spend time foraging [37]. At approximately six weeks of age, pups start to forage with the group (i.e. moving around the territory with them); they are still reliant on adults for food and anti-predator protection until about three to four months of age [38]. Adult group members cooperate to defend a shared territory (mean size = 25 ha; Dwarf Mongoose Research Project 2011–2021, unpublished data) from conspecific rivals; territorial defence involves scent-marking at communal latrines and engagement in IGIs when rivals are encountered [30,31]; further details in section ‘Life-history data collection’.

### (b) Study site and population

Data were collected as part of a long-term study of wild dwarf mongooses, the Dwarf Mongoose Research Project (hereafter 'DMRP') on Sorabi Rock Lodge, a 400 ha private game reserve located in Limpopo Province, South Africa (24°11′ S, 30°46′ E); further details in [39,40]. Daily rainfall is recorded from a rain gauge on the reserve. The DMRP was established in 2011 and has run continuously since, with up to eight mongoose groups habituated to close (less than 5 m) human presence monitored at a time. All individuals are identifiable from unique blonde-hair-dye marks (applied with an elongated paintbrush) or distinct physical features such as scars [39]. The sex of individuals is established by observation of ano-genital grooming [34], and adults (those older than 12 months) are classified as either dominant (the male and female breeding pair) or subordinate (all remaining adults) through observation of agonistic interactions, scent-marking and grooming [39]. There is no sexual size dimorphism in this species [41]. Study groups have a mean ± s.e. adult group size of 8 ± 0.2 (range = 3–17; *n* = 54 group-years of study) [42]. All study individuals are trained to climb onto a balance scale for a small reward, with the aim to collect regular body-mass data, including on first emergence from the sleeping burrow in the morning.

### (c) Life-history data collection

The DMRP maintains a year-round field team of four researchers. Each mongoose group is visited every week for 2–3 days, meaning equivalent data collection across time; individual researchers rotate between all the study groups. The reproductive data used in this study span the breeding season which coincides with the summer months in South Africa (September to March). During this period, the day is split into morning and afternoon observation sessions which are approximately 4 h each. Morning sessions start when the mongooses emerge from their night-time sleeping burrow; evening sessions end when the mongooses enter their night-time sleeping burrow. Observers maintain habituation levels (via their presence and the weighing of mongooses), re-apply dye-marks when they start to fade and track group movement with a GPS. They also collect data on group size and composition, individual and group behaviour (including IGIs) and life-history events (including pregnancies, births and pup emergence from the breeding burrow).

IGIs were recorded ad libitum whenever a focal group encountered a group of conspecific outsiders (*n* = 182 over the breeding seasons). Of these, 94% were with whole groups (171 of 182 IGIs) and 6% occurred with coalitions of individuals (11 of 182 IGIs). Coalitions are usually same-sex individuals either roving to find breeding opportunities or dispersing to find vacancies in other groups. Over the breeding season, a group encounters another group at a mean ± s.e. rate of 1.8 ± 0.2 times per month (range: 0–9). To give this context, banded mongoose groups interact with another group at a rate of 0.6–3 times per month [43]. In dwarf mongooses, IGIs range from signal exchanges (mainly visual and acoustic, 67 of 182 IGIs, 36.8%) to physical encounters (115 of 182 IGIs, 63.2%). Similar to dwarf mongooses, 64.7% of IGIs in meerkats (*Suricata suricatta*) involve physical aggression [14]. Physical encounters in dwarf mongooses involve aggressive chasing by some or all of the individuals in the group; some such encounters escalate into intergroup fighting (44 of 115 events, 38.3%). None of the physical encounters observed over the breeding seasons resulted in any direct mortalities to adults or pups. When an IGI occurred at or near the burrow where the young pups were based (before six weeks of age), the group subsequently moved the pups to a new burrow. When an IGI occurred once the pups had started foraging with the group (approx. six weeks of age onwards), the pups tended not to participate actively (i.e. in chasing and fighting); in these instances, the pups were always in close proximity to the IGI and the adults returned quickly to the pups afterwards. Occasionally pups would also get caught in the melee of IGIs. Consistent data on which group initiated an IGI or the outcome of an interaction were not available and so could not be considered in the analyses.

The pregnancy status of adult females was tracked by monitoring body mass and visible anatomical signs: when pregnant, females exhibit swelling of the abdomen and nipples. The birth of a litter was identified by a sudden reduction in body mass, changes in the visible appearance of the female and changes in group behaviour: once pups are born, subordinate individuals remain at the burrow to babysit while the rest of the group forage, and groups reliably return to the same burrow at the end of the day. Pup emergence was defined as the first time that pups were seen at the burrow entrance, having emerged by themselves. An emerged pup was assumed to have died when it was not present during two consecutive observation sessions.

### (d) Sentinel behaviour data collection

To examine how IGIs and the threat of intergroup conflict influence sentinel behaviour, we conducted observations following both IGIs and latrining events to compare with observations in matched-control periods; data were collected between 2017 and 2019. Latrining involves territorial scent-marking (urinating, defecating, cheek-gland marking and anal-gland marking) at communal sites (rocks, shrubs, termite mounds) where one or more rival groups have also often scent-marked; discovering recent scent-marks from rival groups likely reflects an increased intergroup threat [30,31]. Once the pups had started foraging with the group, they would attempt to participate in the latrine with the adults. Matched-control periods were those of the same duration within 3 days either before or after the IGI or latrine event, at approximately the same time of day and in a similar territory area (within 100 m of a territory border) when group size was the same. We used control periods both before and after IGI and latrine events so that there was no order effect in data collection. During the 60 min period following an IGI and the 30 min period following a latrine event (the longer period for IGIs was chosen as they are more intense events than latrining), and in equivalent control periods (generating observation-period pairs), we recorded all sentinel bouts. Sentinels were defined as individuals positioned on an object (e.g. termite mound, tree, rock), with their hind feet at least 10 cm above the surrounding substrate, and actively scanning the surroundings while groupmates were engaged in other activities, primarily foraging [34]. Individuals younger than 1 year contribute relatively rarely to sentinel behaviour, so data collection and analyses focused on adults. For each bout, we also recorded its duration and the identity of the sentinel.

### (e) Data analysis

We conducted all analyses in R v. 4.1.3 (R Core Team, 2022), building linear mixed models (LMMs) and generalized linear mixed models (GLMMs) using the R package ‘lme4′ [44], except GLMMs with a beta family for which the R package ‘glmmTMB’ was used [45]. For the life-history models, we included group identity and group year as random terms to account for multiple litters from the same group and breeding season. For the sentinel behaviour models, we included the observation-period pair (post-IGI and matched control or post-latrine and matched control) nested within group identity as random effects to account for multiple observation-period pairs from the same group. For both life-history and sentinel-behaviour analyses, we used a global model approach with no model refinement, presenting output values (estimates, standard errors, *z*-values, *t*-values) from the global models. There is an argument for using a model averaging approach when data are observational and collected ad libitum (as for our life-history databases). We therefore also present results from this statistical approach in table S1 of the electronic supplementary material; we find qualitatively the same findings as with the global model approach.

After building an initial global model for a given response variable, based on *a priori* predictions of relevant fixed effects, the model fit was checked by visually inspecting residuals (Pearson residuals for LMMs and deviance residuals for GLMMs) and confirming there was no multicollinearity, using the R package ‘performance’. We also used the function *vif* in the R package ‘car’; variance inflation factors were all less than 3, thus all fixed effects were retained in the global model [46]. For Poisson GLMMs, we also checked for overdispersion. For GLMMs, an appropriate distribution family was used (see below), and the best link function for a given family was chosen based on the best model fit (residual checks and lowest AIC values). For the sentinel behaviour models, logarithmic transformations were conducted to achieve normality in some cases (details below and in electronic supplementary material, table S2), and separate models were run for the post-IGI and post-latrine datasets. All tests were two-tailed and considered significant at *p* < 0.05.

### (f) Life-history data analysis

To examine the relationship between IGI rate and reproductive success, we used data from six breeding seasons between 2012 and 2019 (one breeding season in that period was discarded due to incomplete data collection). We included all types of IGI in our analyses, regardless of who the interaction was with (coalitions or whole groups) and whether it escalated to physical fighting, as all interactions incur at least some costs [27,28]. For each response measure (detailed information provided in the following paragraphs of this section), we assessed the influence of various fixed effects (table 1 for the full list in each case). All models examining reproductive response measures incorporated IGI rate, which was calculated by dividing the number of IGIs a group had over a given period by the number of observation sessions in the same period (an observation session was a 4 h period either in the morning or the afternoon; see section ‘Life-history data collection’). Models also included weighted adult group size and mean adult body mass. Weighted group size was calculated to account for the varying number of adults for different durations over a given period. For instance, if in a time period of 90 days, group size was 8 adults for 30 days, 9 adults for 45 days and 10 adults for 15 days, the weighted group size was 8.83; calculated as ((8 × 30) + (9 × 45) + (10 × 15)) ÷ 90. Mean adult body mass was calculated from all morning body-mass measurements from group members during the relevant period. Where appropriate, we also included: the total amount of rainfall recorded in a relevant timeframe; whether the dominant female lost her first or second litter (yes or no); whether there had been a changeover in the dominant female (yes or no); whether it was the first time that the dominant pair had bred together (yes or no); whether a subordinate female had given birth at the same time as the dominant female (yes or no); and the breeding attempt number in the season (first, second, third).

**Table 1.** Mixed-model outputs for (a) the likelihood of a third breeding attempt (binomial GLMM, logit link), (b) inter-litter interval (LMM), (c) the number of pups surviving to three months post-emergence from the breeding burrow (Poisson GLMM, sqrt link), (d) the number of pups to emerge initially from the breeding burrow (Poisson GLMM, sqrt link), (e) the proportion of pups surviving to three months post-emergence (binomial GLMM, logit link) and (f) the number of intergroup interactions (IGIs) a group had (Poisson GLMM, log link). Group identity and group year were included as random effects (s.d. reported for random effects). Abbreviations: dom, dominant; sub, subordinate. *z*-values are provided for GLMMs, *t*-values for LMMs. For categorical fixed effects with a binary outcome (yes or no), the reference level in the table is ‘no’. Fixed effects in italics if significant.

	effects	estimate ± s.e.	95% CI	*z*	*t*	*p*
(a) likelihood of a third breeding attempt (*n* = 42 group-breeding seasons, 11 groups)						
random effects	group ID	0.00 ± 0.00				
	group year	0.00 ± 0.00				
fixed effects	(intercept)	0.48 ± 1.36				
	IGI rate	9.69 ± 14.67	−19.07, 38.44	0.66	—	0.509
	group size	−0.17 ± 0.16	−0.49, 0.15	−1.07	—	0.286
	dom female changeover	*−2.31 ± 1.18*	*−4.61, −0.01*	*−1.97*	*—*	*0.049*
	lost first litter	0.82 ± 0.90	−0.93, 2.59	0.92	—	0.355
(b) inter-litter interval (*n* = 36 intervals, 10 groups)						
random effects	group ID	0.00 ± 0.00				
	group year	151.55 ± 12.31				
fixed effects	(intercept)	49.38 ± 7.54				
	IGI rate	26.82 ± 62.37	−95.41, 149.06	—	0.43	0.670
	group size	0.11 ± 0.44	−0.76, 0.98	—	0.25	0.805
	rainfall	*0.14 ± 0.03*	*0.08, 0.21*	*—*	*4.42*	*<0.001*
(c) number of pups surviving to three months (*n* = 96 litters, 11 groups)						
random effects	group ID	0.00 ± 0.00				
	group year	0.00 ± 0.00				
fixed effects	(intercept)	2.16 ± 1.92				
	IGI rate	*3.43 ± 1.70*	*0.09, 6.77*	*2.02*	*—*	*0.044*
	group size	*0.05 ± 0.02*	*0.01, 0.10*	*2.26*	*—*	*0.024*
	rainfall	0.00 ± 0.00	−0.00, 0.00	0.06	—	0.953
	first time dom	−0.24 ± 0.14	−0.50, 0.03	−1.74	—	0.082
	sub birth	*−0.32 ± 0.12*	*−0.56, −0.08*	*−2.63*	*—*	*0.009*
	litter no.	−0.05 ± 0.09	−0.22, 0.12	−0.57	—	0.569
	body mass	0.00 ± 0.01	−0.02, 0.01	−0.46	—	0.643
(d) number of pups to emerge (*n* = 94 litters, 11 groups)						
random effects	group ID	0.00 ± 0.00				
	group year	0.00 ± 0.00				
fixed effects	(intercept)	3.72 ± 1.50				
	IGI rate	0.55 ± 1.45	−2.29, 3.39	0.38	-	0.705
	group size	0.02 ± 0.02	−0.03, 0.06	0.71	—	0.478
	rainfall	0.00 ± 0.00	0.00, 0.00	0.57	—	0.571
	first time dom	−0.18 ± 0.13	−0.44, 0.08	−1.35	—	0.177
	sub birth	−0.07 ± 0.12	−0.31, 0.17	−0.59	—	0.559
	litter no.	−0.09 ± 0.11	−0.30, 0.12	−0.87	—	0.387
	body mass	−0.01 ± 0.01	−0.02, 0.00	−1.23	—	0.220
(e) proportion of pups surviving post-emergence (*n* = 93 litters, 11 groups)						
random effects	group ID	0.02 ± 0.15				
	group year	0.00 ± 0.00				
fixed effects	(intercept)	0.22 ± 3.21				
	IGI rate	*9.16 ± 3.46*	*2.39, 15.94*	*2.65*	*—*	*0.008*
	group size	*0.16 ± 0.06*	*0.05, 0.27*	*2.85*	*—*	*0.004*
	rainfall	0.00 ± 0.00	0.00, 0.00	0.12	—	0.904
	first time dom	−0.20 ± 0.33	−0.84, 0.44	−0.61	—	0.542
	sub birth	*−1.20 ± 0.30*	*−1.79, −0.61*	*−4.00*	*—*	*0.004*
	litter no.	0.02 ± 0.27	−0.51, 0.55	0.07	—	0.941
	body mass	0.00 ± 0.01	−0.03, 0.02	−0.21	—	0.832
(f) number of intergroup interactions (*n* = 96 post-emergence periods, 11 groups)						
random effects	group ID	0.14 ± 0.37				
	group year	0.12 ± 0.35				
fixed effects	(intercept)	−0.08 ± 2.28				
	group size	0.00 ± 0.04	−0.07, 0.07	0.07	—	0.946
	rainfall	*−0.00 ± 0.00*	*−0.01, −0.00*	*−2.87*	*—*	*0.004*
	body mass	−0.01 ± 0.01	−0.03, 0.00	−1.35	—	0.177

Initially, we used separate models to investigate the relationship between IGI rate and (i) the number of breeding attempts by dominant females in a breeding season, (ii) inter-litter interval and (iii) the number of pups alive at three months post-emergence from the breeding burrow. We defined each breeding season as the period between the date at which the first dominant female in the study population came into oestrous (when she was seen repulsing advances from males or mating) and the date when the last litter of pups in the study population emerged. For analysis of the number of breeding attempts, IGI rate and weighted group size were calculated for the full breeding season. Apart from one group in one breeding season, dominant females always had a minimum of two breeding attempts and a maximum of three. We therefore analysed breeding attempts in a binomial GLMM with a logit link function, asking whether IGI rate was associated with the likelihood of having more than two breeding attempts (yes or no); we excluded the data point from the group that had only one litter in a breeding season (for analysis, *n* = 42 group-breeding seasons, 11 groups). For analysis of inter-litter interval, we ran an LMM considering the period between the birth of the first and second litters in a season. IGI rate, weighted group size, mean adult body mass and rainfall were calculated over the inter-litter period for each group in each breeding season. Before running the model, we removed four occasions where there had been a changeover in the dominant female as this heavily skewed the inter-litter interval (for analysis, *n* = 36 intervals, 10 groups). For analysis of the number of pups alive at three months post-emergence, we ran a GLMM with a Poisson error distribution and a square-root link. IGI rate, weighted group size, mean adult body mass and rainfall were calculated for each litter over the period from when the dominant female became pregnant (established by subtracting 54 days, the gestation period in dwarf mongooses [47], from the day the litter was born) until 90 days after the first emergence of the pups from the breeding burrow. The analysis was conducted on 96 litters from 11 groups.

Having found a significant relationship between IGI rate and the number of pups alive at three months post-emergence (see Results and table 1*c*), we used separate models to investigate whether this was due to a difference in (i) the number of pups first emerging from the burrow and/or (ii) the proportion of pups surviving post-emergence. For analysis of the number of pups to emerge, we used a GLMM with a Poisson error distribution and a square-root link. IGI rate, weighted group size, mean adult body mass and rainfall were calculated for each litter over the period from when the dominant female became pregnant (see above) to when the pups first emerged from the burrow. We excluded two litters due to uncertainty about the exact day of emergence (for analysis, *n* = 94 litters, 11 groups). For analysis of the proportion of pups surviving post-emergence, we used a GLMM with a binomial error distribution and a logit link function. The model bound the number of emerged pups that survived (successes) with the number of emerged pups that died (failures). IGI rate, weighted group size, mean adult body mass and rainfall were calculated over the three month post-emergence period. We excluded three litters that had no pups emerging from the burrow (for analysis, *n* = 93 litters, 11 groups).

To assess further the potential importance of group size and adult body mass in explaining the relationship between IGI rate and post-emergence pup survival, we analysed factors affecting the number of IGIs a group had in a GLMM with a Poisson error distribution and a log link function. Number of IGIs, weighted group size, mean adult body mass and rainfall were calculated over the three month post-emergence period. The model included an offset term (log number of observation sessions) to account for differences in the number of observation sessions. The analysis was conducted on 96 post-emergence periods from 11 groups.

### (g) Sentinel behaviour data analysis

Initially, we analysed whether observation period (control versus either post-IGI or post-latrine) affected the proportion of time that individuals acted as a sentinel. Since a significant effect was found in both cases (see Results and table 2), we analysed whether these overall effects were driven by differences in (i) sentinel occurrence rate, (ii) the proportion of adult group members engaging in sentinel behaviour and/or (iii) mean sentinel bout duration. To allow use of the beta family in GLMMs, the single zero value in the IGI dataset (due to no individuals displaying sentinel behaviour) was changed to 0.001 for analyses of the proportion of time, sentinel occurrence rate and the proportion of the group engaged, and to 1 second for analysis of the mean sentinel bout duration (to enable log-transformation). In one observation period, all individuals were involved in sentinel behaviour; this proportion of 1 was changed to 0.999. These changes probably had little effect but would, if anything, make our results more conservative.

**Table 2.** Mixed-model outputs investigating the effects of (a) intergroup interactions (IGIs) and (b) latrine activity on the proportion of time that sentinel behaviour occurred compared to matched-control periods. Both models included observation period (I, post-IGI; C, control; L, post-latrine) as a fixed effect (in italics if significant), and observation-period pair nested within group ID as random effects. *z*-values are provided for the GLMM, *t*-values for the LMM, s.d. reported for random effects. The reference level for the fixed effect of observation period is ‘control’.

	effects	estimate ± s.e.	95% CI	*z*	*t*	*P*
IGIs (*n* = 18 paired observation periods)						
(a) proportion of time sentinel behaviour occurred (beta GLMM, logit link)						
random effects	period pair in group ID	0.00 ± 0.00				
	group ID	0.19 ± 0.43				
fixed effects	(intercept)	−2.13 ± 0.32				
	observation period	*0.80 ± 0.30*	*0.21, 1.39*	*2.66*	*—*	*0.008*
latrines (*n* *=* 49 paired observation periods)						
(b) proportion of time sentinel behaviour occurred (LMM with log-transformed data)						
random effects	period pair in group ID	0.06 ± 0.25				
	group ID	0.04 ± 0.21				
fixed effects	(intercept)	−2.39 ± 0.19				
	observation period	*0.70 ± 0.23*	*0.25, 1.16*	*—*	*3.02*	*0.004*

## 3. Results

### (a) Life-history data

**Figure 1.**
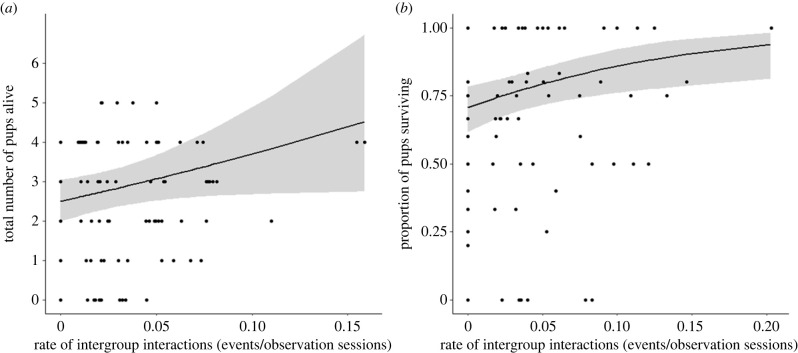
The relationship between intergroup interaction (IGI) rate and (*a*) the total number of pups alive at three months post-emergence (*n* = 96 litters from 11 groups) and (*b*) the proportion of post-emergence pups surviving to three months (*n* = 93 litters from 11 groups). The IGI rate is calculated from when the dominant female became pregnant until 90 days after the first emergence of the pups from the breeding burrow in (*a*) and over the three month post-emergence period in (*b*); see Methods for details. The solid lines show predictions from the GLMMs with 95% confidence intervals shown as grey bands.

Using data from nearly 100 litters produced by 11 wild dwarf mongoose groups across six breeding seasons, we found a positive relationship between IGI rate and reproductive success. Groups produced up to three litters per season (mean ± s.e. = 2.3 ± 0.1; *n* = 42 group-breeding seasons of 11 groups) but there was no significant effect of IGI rate on the likelihood of a group having a third litter (GLMM: *z* = 0.66, *p* = 0.509; table 1*a*). There was also no significant effect of IGI rate on the inter-litter interval (mean ± s.e. = 71 ± 2 days, range: 46–94 days; *n* = 36 intervals from 10 groups; LMM: *t* = 0.43, *p* = 0.670; table 1*b*). However, there was a positive correlation between IGI rate and the number of pups that were alive three months after first emerging from the breeding burrow (mean ± s.e. = 2.4 ± 0.2, range: 0–5; *n* = 96 litters from 11 groups; GLMM: *z* = 2.02, *p* = 0.044; table 1*c* and figure 1*a*).

A difference in the number of pups present three months post-emergence could be because of a difference in the number born, survival rate before emergence from the breeding burrow and/or survival rate post-emergence. The positive relationship between IGI rate and pup number at three months was not because significantly more pups initially emerged from the burrow (mean ± s.e. = 3.6 ± 0.2, range: 0–6; *n* = 94 litters from 11 groups; GLMM: *z* = 0.38, *p* = 0.705; table 1*d*). Thus, there was no evidence that IGI rate affected reproductive success in terms of the number of pups born or how well they survived in the first three weeks of life spent entirely underground. Instead, IGI rate was positively correlated with pup survival post-emergence (mean ± s.e. proportion surviving = 0.66 ± 0.03, range: 0–1; *n* = 93 litters from 11 groups; *z* = 2.65, *p* = 0.008; table 1*e* and figure 1*b*). Therefore, IGI rate was related to how well pups survived once they were spending time above ground during the day.

There was no evidence that group size (GLMM: *z* = −0.07, *p* = 0.946) or adult body mass (*z* = −1.35, *p* = 0.177) was related to the number of IGIs a group had (table 1*f*).

### (b) Sentinel behaviour

**Figure 2.**
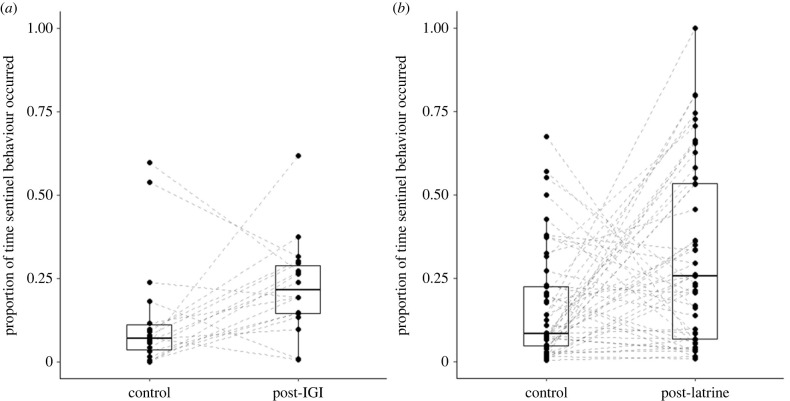
The proportion of time that sentinel behaviour occurred in the (*a*) 60 min post-IGI and (*b*) 30 min post-latrine observation periods compared to matched-control periods of the same duration (*n* = 18 and 49 period pairs, respectively). Box plots show medians and quartiles, whiskers show upper and lower quartiles (±1.5 times the interquartile range), and dashed lines join data points from matched observation periods (black dots).

Using data from matched observation periods, we found that sentinel behaviour (raised guarding) occurred a greater proportion of the time following an IGI than in control periods (GLMM: *z* = 2.66, *p* = 0.008; table 2*a* and figure 2*a*). Moreover, after time spent at communal latrines, where there are likely scent-marks indicating the recent presence of a rival group, a similarly greater occurrence of sentinel behaviour compared with control periods was detected (LMM: *t* = 3.02, *p* = 0.004; table 2*b* and figure 2*b*). In both scenarios, the greater proportion of time engaged in sentinel behaviour was driven, at least in part, by an increase in the rate of sentinel occurrence (GLMM, IGIs: *z* = 2.40, *p* = 0.016; electronic supplementary material, table S2*a*; latrines: *z* = 2.87, *p* = 0.004; electronic supplementary material, table S2*d*). More sentinel time following latrine behaviour was also the consequence of a greater number of group members acting as a sentinel (*z* = 2.41, *p* = 0.016; electronic supplementary material, table S2*e*) and sentinel bouts being longer in duration (LMM: *t* = 2.04, *p* = 0.047; electronic supplementary material, table S2*f*).

## 4. Discussion

Outgroup contests, especially those that escalate to physical violence, are well known to have substantive immediate and delayed fitness consequences [10]. By showing that dwarf mongoose pups survived better when groups were more frequently engaged in IGIs, we add to the small body of evidence that the cumulative threat of outgroup conflict can also affect reproductive output [27–29]. The conflict-related improvement in dwarf mongoose pup survival is not counteracted by more infanticide or abortion, as these latter events occur only very rarely in our study population: infanticide has been observed only once, and abortion only three times, in more than 120 breeding attempts (DMRP 2011–2021, unpublished data). Our study therefore highlights the possibility that conflict with rival groups could have positive effects, not just the negative reproductive consequences previously documented [27–29]; better offspring survival might arise as a by-product of conflict-induced increases in vigilance behaviour. Moreover, it showcases the need to think beyond individual contests and to assess cumulative threat when considering outgroup conflict as a social driver of fitness.

Our reproductive success results are correlative—as we accumulated ecologically valid data from the long-term study of a wild population—so, in principle, the positive association between IGI rate and pup survival could be due to a confounding effect of group size and/or body condition. For example, larger groups or those with individuals in better condition might be better able to raise pups successfully and to engage in more IGIs. However, group size and body mass were included as fixed effects in our models showing a significant effect of IGI rate on pup survival (table 1*c,e*), and separate modelling found no evidence that group size or adult body mass is related to the likelihood of a group having an IGI (table 1*f*). Another possibility is that participation in more IGIs prevents raids by rival groups seeking to kill offspring; raiding behaviour is seen in, for example, banded mongooses [27]. But that is unlikely to be an explanation for our findings because dwarf mongooses have only been observed to engage in such infanticidal behaviour once in more than a decade of study (DMRP 2011–2021, unpublished data). We also do not believe that pup survival improves because of some direct positive effect of interactions with rival groups. Rather, our behavioural data suggest that one potential reason for better pup survival once they have emerged from the breeding burrow is that outgroup threat results in increased adult vigilance.

It is now well established that outgroup conflict can lead to a range of behavioural changes [48–54]. Our analyses in this paper show that adult dwarf mongoose sentinel behaviour—raised guarding where an individual is dedicated to vigilance—increases in the aftermath of both IGIs and latrine events. Such behavioural changes in response to even just the interaction with rival-group cues (e.g. faecal samples) can potentially last beyond the immediate aftermath of the interaction and into the following day [53]. Since dwarf mongoose IGIs and latrine events occur regularly (mean ± s.e. rate = 0.14 ± 0.01 occurrences per hour, range: 0.04–0.28; *n* = 42 group-breeding seasons of 11 groups), intergroup-related increases in sentinel behaviour are likely relatively frequent. While doing more sentinel behaviour on such occasions may be an attempt by individuals to gain information about the presence of conspecific rivals, sentinels are more likely than foragers to spot predators and to give alarm calls to warn of such threats [55]. The increase in this vigilance behaviour could therefore, in principle, lessen the predation risk to vulnerable offspring; pups are reliant on adults for protection from a variety of terrestrial and aerial predators until approximately three to four months of age [38]. Pups are often with the adults following an IGI and during a latrine and so would benefit from this increased vigilance (details in section ‘Life-history data collection’). Future work is, however, required to demonstrate a causal link. This would involve analysis of how outgroup conflict affects vigilance over long periods and, ultimately, fitness; our reproductive and vigilance analyses are over different timeframes, and we do not have consistent sentinel data across the whole breeding season due to the density of vegetation.

In contrast to previous studies of banded mongooses, chimpanzees and cichlid fish, which report negative effects of outgroup conflict on reproductive success [27–29], and a study of Tasmanian native hens (*Tribonyx mortierii*) that found no effect [56], our results indicate the possibility of a positive impact. Such interspecific variation might be related to differences in, for instance, reproductive ecology (e.g. seasonal versus year-round breeding), the frequency and intensity (e.g. escalation level) of outgroup encounters, the foraging and predation pressures faced, and how outgroup conflict influences within-group social behaviour and relationships [11]. More generally, positive as well as negative effects may arise from the same outgroup contest or accumulation of threat; such differences may occur between groups or individuals within the same group [11]. There might also be variation in reproductive consequences between groups related not just to IGI rate (as tested in this study) but, for instance, IGI initiation, type and outcome of a contest and relative resource-holding potential or group size. Establishing the full range of fitness costs and benefits, and reasons for the variation both within and between species, is important because intergroup conflict is believed to be a powerful selective pressure in the evolution of, for example, cognitive abilities, group dynamics and social structure [7–9]. Therefore, as the empirical evidence for a diverse range of fitness consequences increases, it is important that theoretical modelling incorporates these different drivers to assess their likely impact on the evolutionary significance of intergroup conflict.

## Data Availability

Data are available from Dryad Digital Repository [57] and are provided in the electronic supplementary material [58].
